# Systematic Pan-Cancer Population-Based Analysis Reveals the Incidence and Prognosis of Lung Metastases at Diagnosis

**DOI:** 10.1155/2021/9999968

**Published:** 2021-06-15

**Authors:** Xiaohong Liang, Yinan Cheng, Weijun Zhou, Jun Ni, Yuqing Li, Gaohua Feng

**Affiliations:** Department of Pulmonary and Critical Care Medicine, Zhangjiagang TCM Hospital Affiliated to Nanjing University of Chinese Medicine, No. 4, Kangle Road, Zhangjiagang City, Jiangsu Province, China

## Abstract

**Background:**

Metastasis is one of the most prevalent causes of death in cancer patients and the lungs are among the organs most commonly affected by metastasis. However, analysis of the incidence and prognosis of lung metastasis (LM) based on primary cancer sites is lacking.

**Methods:**

We enrolled cancer patients with LM from the Surveillance, Epidemiology, and End Results (SEER) database. The risk factors for LM were determined using multivariate logistics regression. Forest plots were used to compare the impact of with LM versus without LM alone among different primary caner site subgroups.

**Results:**

Among 1,525,441 cases, 47,537 presented with LM at initial diagnosis. Multivariate logistics regression revealed that male sex, older age, later T/N stage, unmarried status, and lack of insurance were risk factors for LM. The incidence of LM was 11.91% in bone cancer and 11.19% in pancreatic cancer. In terms of the distribution of primary cancers, 19.22% of LMs originated from the colon and rectum, with 11.63% from the kidneys. The median survival for LM cases was 6 months, with the best survival in testicular cancer (19 months) and bone cancer (12 months). Patients with LM had higher hazard ratio (HR) for mortality compared to those without LM, except for those with primary cancer in the brain (*P*=0.09). We stratified patients by primary cancer site, and subgroup analyses showed that LM had a significant negative impact on survival. The most significant was in thyroid cancer (HR = 44.79), followed by melanoma (HR = 24.26), prostate (HR = 16.0), breast (HR = 13.46), endometrial (HR = 12.64), testicular (HR = 12.31), and kidney (HR = 11.33) cancer (all *P* < 0.001).

**Conclusion:**

Patients presenting with LM had higher HR for mortality compared to those without LM, except for those with brain tumor. Clinicians should pay more attention to the occurrence of LM, especially in patients with a significantly increased HR for mortality, such as those with thyroid cancer, melanoma, and prostate cancer.

## 1. Background

Cancer has been one of the main global health problems over the years and so far has been the second primary public death cause. In 2020, it was reported that there were 1,806,590 new cancer cases and 606,520 cancer deaths in the USA [1]. Metastatic disease represents the most prevalent cause of cancer-related death [2]. Among the vital organs to which solid tumors metastasize, the lungs are among the most commonly affected, ranging from 20% to 45% [3].

Due to the ongoing development of new treatments and better survival outcomes over the years, the trend may continue to increase. Most lung metastasis (LM) is detected incidentally without symptoms or with nonspecific symptoms. Treatment of LM requires a comprehensive approach. Surgical resection can be performed in suitable cases. Good survival outcomes have been demonstrated in patients with many different primary cancers who underwent pulmonary metastasectomy [4]. Data from the International Registry of Lung Metastases show that, for various histological types of tumors, the 5-, 10-, and 15-year survival rates after complete resection of metastases were 36%, 26%, and 22%, respectively [4]. If LM is not suitable for surgical resection, radiotherapy and chemotherapy can be used.

Different tumors have their own proclivity to metastasize to target organs. LM is most common from primary breast, colon, prostate, and bladder cancers, neuroblastoma, and sarcoma [5]. When cancer cells leave their primary site and migrate to the lungs, they change from quiescence into metastatic outgrowth and infiltrate surrounding tissues. At the same time, the environment of local blood flow, hypoxia, and inflammation in the lungs accelerates this pathophysiological process. LM develops due to various mechanisms, including local blood flow and cellular or biochemical properties of the tumor cells. Although LM and its associated mortality have a high incidence, the potential mechanisms by which metastatic tumor cells grow and thrive in the lung microenvironment remain elusive.

Many of the current studies [6–9] on LM are on single primary tumors, and a generalized analysis of LM is lacking. In the current study, we used the Surveillance, Epidemiology, and End Results (SEER) database to perform a comprehensive analysis to check the status of LM. The study was designed to show a generalizable recognition of the incidence and prognosis of LM across multiple types of cancer. We also analyzed the epidemiological trends by clinicopathological factors to explain the potential disparities among patients, including biological factors (age, sex, T stage, and N stage) and socioeconomic factors (race, insurance, and marriage). These data may assist in clinical decision-making concerning lung-specific surveillance and provide epidemiological evidence for the estimation of disease burden for both policy-makers and healthcare service providers.

## 2. Materials and Methods

### 2.1. Data Source

Our study was based on the SEER cancer registry, which is the population-based registry for cancer incidence in the USA and a publicly available and reliable database. All the information was obtained using SEER*∗*Stat version 8.3.8. Currently, it documents the cancer cases from 18 registry sites, encompassing roughly 28% of the general population in USA. After obtaining permission, the research files were extracted from the SEER database and there was no need for informed consent.

### 2.2. Patient Selection

We enrolled patients diagnosed with only one primary cancer using positive histology between January 1, 2010, and December 31, 2016. Cases that originated in the lungs and those with unknown LM status were excluded, along with cases without survival information. Patients diagnosed with T0 or Tis stage based on the American Joint Committee on Cancer (AJCC) 7th TNM staging system and cases within situ were also removed. In addition, the SEER database provides clinical variables, including age at diagnosis, sex, race, T and N stages, race, marital status, instance, and primary cancer sites. Overall survival (OS) time was obtained from the SEER database. Age at diagnosis was categorized into three groups: young (≤45 years); middle-aged (45–75 years); and elderly (≥76 years).

### 2.3. Statistical Analysis

The numbers of overall cases, metastatic cases, and cases with LM were summarized according to primary cancer, with median survival and interquartile range for LM cases. Survival analysis was performed using Kaplan–Meier curves. Additionally, the crude incidences of LM and the incidence of LM overall metastases were calculated. A multivariate logistics regression model was applied to filter out risk factors for cancer patients with LM. Forest plots were used to compare the impact of with lung metastases versus without lung metastases alone among different primary caner sites subgroups. All data analysis was performed using SPSS 23.0 (IBM Corporation, Armonk, NY, USA). All statistical tests were performed two-sided. *P* < 0.05 was considered to be statistically significant.

## 3. Results

### 3.1. Incidence of LM Based on Clinicopathological Characteristics

We enrolled 1,525,441 eligible patients with LM diagnosed between 2010 and 2016 from the SEER database. The basic characteristics of cancer patients with or without LM are depicted in Table 1. Among 1,525,441 patients, 47,537 (3.12%) had LM at initial diagnosis. The incidence of LM in elderly, middle-aged, and young patients was 3.72%, 3.01%, and 2.95%, respectively. The incidence of LM was higher in men than in women (3.23% versus 3.01%). From T1 to T4 stage, patients with later stages had a higher incidence of LM (0.79%, 2.02%, 4.31%, and 9.77%, respectively). The incidence of LM in patients with N0, N1, N2, and N3 subtypes was 1.54%, 6.70%, 5.25%, and 7.77%, respectively. The incidence of LM in insured versus uninsured patients was 3.15% versus 6.0%. The incidence of LM in unmarried versus married patients was 4.14% versus 2.69%.

### 3.2. Risk Factors for Developing LM Based on Multivariate Logistic Regression

Factors associated with LM formation included age, sex, race, marital status, insurance status, T stage, and N stage (Table 2). Multivariate logistic regression analysis showed that younger patients (odds ratio (OR): 0.92, 95% confidence interval (CI): 0.89–0.96; *P* < 0.001), male sex (OR: 1.04, 95% CI: 1.02–1.06; *P* < 0.001), later T stage (OR: 4.48, 95% CI: 4.31–4.66; *P* < 0.001), later N stage (OR: 2.01, 95% CI: 1.89–2.15; *P* < 0.001), Caucasian (OR: 0.93, 95% CI: 0.90–0.96; *P* < 0.001), uninsured (OR: 1.34, 95% CI: 1.27–1.40; *P* < 0.001), and unmarried (OR: 1.28, 95% CI: 1.25–1.31; *P* < 0.001) were significantly positively associated with LM at initial diagnosis.

### 3.3. Incidence of LM Based on Primary Cancer Sites

**Figure 1:**
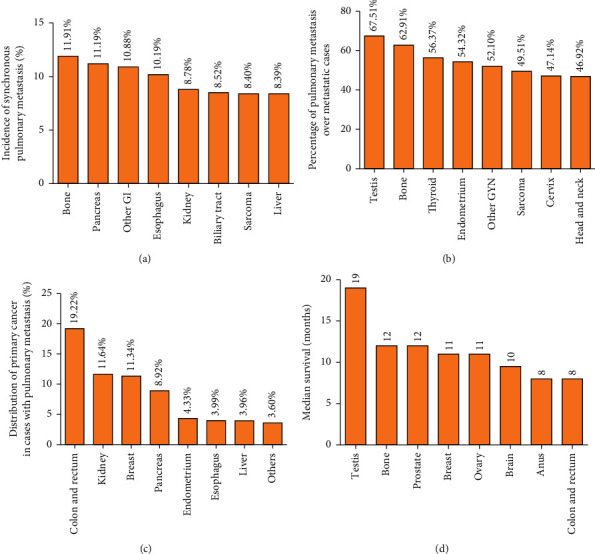
Prevalence and prognosis of LM according to primary cancer type. (a) Incidence of synchronous LM in different cancer types in all cancer patients (including metastatic and nonmetastatic cancer). (b) Incidence of synchronous LM in different cancer types in patients with metastatic lesions. (c) Distribution of primary cancer types in patients with LM. (d) Median survival of cancer patients with LM.

Patients with LM have different primary cancer sites. The leading eight incidences of LM were observed in bone cancer (11.91%), pancreatic cancer (11.19%), other gastrointestinal cancer (10.88%), esophageal cancer (10.19%), kidney cancer (8.78%), biliary tract cancer (8.52%), sarcoma (8.40%), and liver cancer (8.39%) (Figure 1(a) and Table 3). The lungs are the major site of metastasis from testicular cancer (67.51%), bone cancer (62.91%), thyroid cancer (56.37%), endometrial cancer (54.32%), other gynecological cancer (52.10%), sarcoma (49.51%), cervical cancer (47.14%), and head and neck cancer (46.92%) (Figure 1(b) and Table 3). The four most common primary cancers were colorectal (19.22%), kidney (11.64%), breast (11.34%), and pancreatic (8.92%) cancer, accounting for >50% of all LMs. Moreover, 4.33% of LM cases originated from the endometrium, 3.99% from the esophagus, 3.96% from the liver, and 3.60% from other organs (Figure 1(c) and Table 3).

### 3.4. Survival Analysis

The median survival for overall LM cases was 6 months, with the best survival in testicular cancer (19 months), followed by bone (12 months), prostate (12 months), breast (11 months), ovarian (11 months), brain (10 months), anal (8 months), and colorectal (8 months) cancer (Figure 1(d) and Table 3).

**Figure 2:**
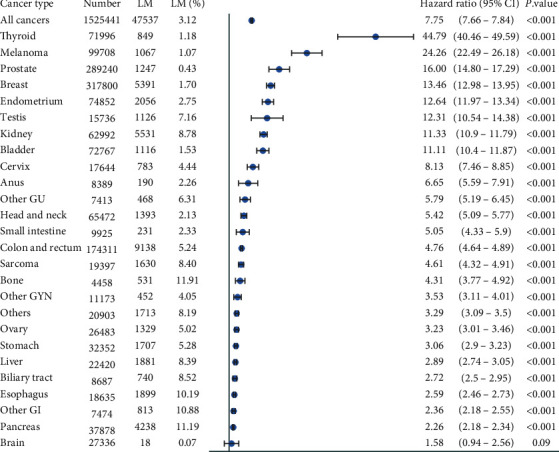
Forest plots depict OS among primary cancer patients with or without LM. GI: gastrointestinal cancer; GYN: gynecological cancer; GU: genitourinary cancer.

Univariate Cox regression analysis compared the hazard ratios (HRs) for mortality in patients with or without LM. Patients with LM had poorer OS compared to those without LM (HR = 7.75; *P* < 0.001) (Figure 2). A Cox regression model was used to analyze HR and 95% CI stratified by primary cancer site. For most primary cancer sites, LM had a significant negative impact on OS, except for patients with primary brain cancer (*P*=0.09) (Figure 2). Forest plots showed that the following primary cancer sites had the greatest negative influence on OS: thyroid (HR = 44.79; *P* < 0.001), melanoma (HR = 24.26; *P* < 0.001), prostate (HR = 16.0; *P* < 0.001), breast (HR = 13.46; *P* < 0.001), endometrium (HR = 12.64; *P* < 0.001), testis (HR = 12.31; *P* < 0.001), and kidney (HR = 11.33; *P* < 0.001).

## 4. Discussion

In the current study, our data were obtained from the SEER database, which covers ∼28% of the general population in USA; the demonstrated trends are of great representativeness and generalizability. We described the basic clinical characteristics of cancer patients with or without LM and explored the risk factors for developing LM. Multivariate logistic regression revealed that male sex, older patients, later T/N stage, unmarried status, and lack of insurance were the independent risk factors for LM. These risk factors may help customize lung monitoring and clinical decision-making.

We performed a comprehensive analysis of the incidence and prognosis of LM at initial diagnosis, according to primary cancer site. LM has been shown to originate most often from breast, followed by rectum, cervix, and stomach [5]. In a study of 5,206 patients who underwent lung metastasectomy, ∼43% of LMs were from epithelial cell tumors, sarcomas (42%), germ cell tumors (7%), and melanoma (6%) [4]. 4572 patients were enrolled from the International Registry of Lung Metastases. Survival analysis stratified by pathological patterns showed that the 5-year survival rate was 36% in patients with epithelial tumors, and 10-year survival rate was 26% in patients with sarcomas. Our research showed that LM was most commonly from colorectal cancer, followed by kidney, breast, and pancreatic cancer.

Our results showed that median OS for LM cases overall was 6 months, with the best for testicular cancer, followed by bone, prostate, breast, ovarian, brain, anal, and colorectal cancer. We found that patients with LM had poorer OS compared to those without LM. In the subgroup analysis, LM from brain cancer did not affect prognosis. For most primary tumors, the risk of mortality is significantly increased when LM occurs. Mortality was increased most significantly for LM from thyroid cancer, melanoma, and prostate, breast, endometrial, testicular, and kidney cancer. Therefore, for clinicians, early detection of LM is important for clinical practice to reduce the risk of mortality and disease burden in cancer patients.

Colorectal cancer was the most common primary cancer for LM in our study, which deserves more attention. For patients with colorectal cancer, the liver and lungs were top two metastatic sites. A previous study [10] has described the pattern of distant metastases in colorectal cancer. Rectal cancer had a higher incidence of LM compared to colon cancer, which is similar to our results, especially for N1 stage rectal cancer. Similar results have been reported in stage IV colorectal cancer [11]. Compared to patients with metastases in other organs such as the liver, bone, and brain, patients with only LM have better OS, specifically among those with KRAS mutant tumors [12]. In patients with LM, colorectal cancer cells usually initially enter the general circulation, followed by infiltration of the lung parenchyma, killing the lung capillary cells. This process involves parathyroid hormone-like hormone (PTHLH) and possibly necroptosis. Chemokines derived from exosomes may promote colorectal cancer cell metastasis to the lungs [9]. Colorectal cancer cells, via a macrophage-dependent pathway, release microparticles to remodel the lung parenchyma and create an altered inflammatory and mechanical response to tumor cell invasion [13]. The specific mechanism of colorectal cancer metastasis to the lungs is still unknown.

Breast cancer is the most common malignant disease in women [1]. More importantly, breast cancer had one of the highest incidences of LM in our research. LM significantly increased the mortality of breast cancer patients. A previous study demonstrated that most breast-cancer-related deaths were related to the occurrence of metastases [14]. Usually decades after initial diagnosis of primary cancer, over half of breast cancer patients will develop metastases, which severely affects disease management [15]. Besides bone, the lungs are the most frequent site of metastasis in breast cancer patients. We identified 317,800 breast cancer patients with LM, representing 1.69% of the entire cases and 24.4% of the subgroup with metastases. This is similar to a recent research [8]. Clinical research has shown that different molecular characteristics affect organ-specific metastatic patterns. HR^−^/HER2^+^ and triple-negative breast cancer patients have the highest incidence of metastasis. Triple-negative patients have poor survival and usually respond poorly to hormonal therapy [16]. Recently, more attention has been paid to the underlying molecular mechanisms that play an important role in the organ-specific metastasis of breast cancer cells [15].

Previous studies have demonstrated that the liver, lung, bone, brain, and distant lymph nodes are the common sites of metastases from pancreatic adenocarcinoma [17–20]. In 19.9% of patients, LMs were found at initial diagnosis of primary cancer. Compared to liver metastasis, patients with LM have better OS. In renal cell carcinoma patients, the lungs are the most common site of metastasis [21, 22]. Surgical intervention may improve OS significantly in renal cell carcinoma patients with LM [6].

The above tumors are more prone to LM, which is accompanied by a complicated pathological process. The process of tumor metastasis is not randomly generated and is regulated by multiple factors, including microenvironmental, cellular, and molecular factors. Circulation patterns are important for circulating tumor cells (CTCs) to mechanically arrest in the capillary networks they encounter. Due to its high vascular flow, hypoxia, and inflammation, the lungs are an example of the most common metastatic targets, with ∼10.7% of all primary malignancies targeting this site [5]. When tumor cells leave their primary site and enter the venous drainage of the organ, it is likely that the first metastatic foci will develop in the filtering organ.

When the tumor grows malignantly *in situ*, it begins to infiltrate the surrounding tissues and enters the circulation through the blood vessel walls to form CTCs [2]. These CTCs reach the target before the metastasis of the organ, the metastasis organ will first form the premetastasis microenvironment, and the primary tumor secretes a large number of factors, including exosomes [23, 24], cytokines [2], and chemokines [9], which recruit bone-marrow-derived cells to metastatic organs and change the extracellular matrix environment of the metastatic organs. The arrival of CTC transplantation provides a living soil. How the factors secreted by these malignant tumor cells affect the normal lung tissue and how they promote LM in the premetastasis microenvironment are still unclear.

Although the current study had some novel findings, it still had some limitations. First, we only obtained qualitative information about LM status from the SEER database rather than specific quantitative information for LM lesions, including the number and size of lesions. Second, the lack of molecular-related information made it impossible to further analyze the relevant mechanisms of LM. Third, underestimation of incidence may occur in cancer types in which routine LM screening is not adopted.

## 5. Conclusion

The current study provided generalizable and representative epidemiological data for LM according to clinicopathological factors and primary cancer sites. Multivariate logistic regression revealed that male sex, older patients, later T/N stage, unmarried status, and lack of insurance were risk factors for LM. We found that patients with LM had higher HR for mortality compared to those without LM, except for brain cancer patients. Clinicians should pay more attention to the occurrence of LM, especially for those with a significantly increased HR for mortality, such as thyroid cancer, melanoma, and prostate cancer. These data not only help medical practitioners tailor screening protocols and design clinical trials but also give an approximation of the public disease burden for policy-makers.

## Figures and Tables

**Table 1:** Clinicopathological characteristics of new diagnosis cancer patients with and without lung metastases.

Patients' characteristics	No. of new diagnosis cancer patients				Total
	With lung metastases		Without lung metastases		*N*
	*N*	%	*N*	%	
All patients	47537	3.12	1477904	96.88	1525441

*Age at diagnosis*					
Elderly	9132	3.72	236053	96.28	245185
Middle-aged	32581	3.01	1050441	96.99	1083022
Young	5824	2.95	191410	97.05	197234

*Sex*					
Male	24004	3.23	718610	96.77	742614
Female	23533	3.01	759294	96.99	782827

*T*					
T1	5392	0.79	675382	99.21	680774
T2	7368	2.02	357589	97.98	364957
T3	10393	4.31	230908	95.69	241301
T4	9035	9.77	83432	90.23	92467
Unknown	15349	10.52	130593	89.48	145942

*N*					
N0	16812	1.54	1074009	98.46	1090821
N1	15184	6.70	211489	93.30	226673
N2	3982	5.25	71829	94.75	75811
N3	1493	7.77	17710	92.23	19203
Unknown	10066	8.91	102867	91.09	112933

*Race*					
White	36239	3.01	1167620	96.99	1203859
Black	6641	3.90	163747	96.10	170388
Others	4491	3.68	117476	96.32	121967
Unknown	166	0.57	29061	99.43	29227

*Insurance*					
Insured	44285	3.15	1359405	96.85	1403690
Uninsured	2362	6.00	36998	94.00	39360
Unknown	890	1.08	81501	98.92	82391

*Marital status*					
Married	22493	2.69	812320	97.31	834813
Unmarried	22842	4.14	529150	95.86	551992
Unknown	2202	1.59	136434	98.41	138636

**Table 2:** Multivariable logistic regression for the presence of lung metastases at diagnosis by cancer type.

Categories		OR (95% CI)	*p*
Age	Elderly	Ref	
	Middle-aged	0.90 (0.87–0.92)	＜0.001
	Young	0.92 (0.89–0.96)	＜0.001

Sex	Female	Ref	
	Male	1.04 (1.02–1.06)	＜0.001

T	T1	Ref	
	T2	1.93 (1.86–2.01)	＜0.001
	T3	3.01 (2.91–3.12)	＜0.001
	T4	4.48 (4.31–4.66)	＜0.001
	Unknown	4.57 (4.40–4.76)	＜0.001

N	N0	Ref	
	N1	1.91 (1.86–1.96)	＜0.001
	N2	1.43 (1.38–1.49)	＜0.001
	N3	2.01 (1.89–2.15)	＜0.001
	Unknown	1.50 (1.45–1.55)	＜0.001

Race	Black	Ref	
	White	0.93 (0.90–0.96)	＜0.001
	Others	1.08 (1.03–1.06)	＜0.001
	Unknown	0.37 (0.32–0.44)	＜0.001

Insurance	Insured	Ref	
	Uninsured	1.34 (1.27–1.40)	＜0.001
	Unknown	0.60 (0.56–0.65)	＜0.001

*Marital status*	Married	Ref	
	Unmarried	1.28 (1.25–1.31)	＜0.001
	Unknown	0.86 (0.82–0.91)	＜0.001

**Table 3:** Numbers of all cases, metastatic cases, and cases with pulmonary metastasis and incidence, distribution, and prognosis of lung metastasis by cancer type.

Categories		Numbers of cases			Incidence (%)		Distribution (%)	Median survival with IQR
Site	Subtype	All	Metastasis	LM	LM	LM/metastasis	Distribution of LM cases	LM
Brain	Brain	27336	280	18	0.07	6.43	0.04	10 (3–20)
Head and neck	Head and neck	65472	2969	1393	2.13	46.92	2.93	7 (3–15)
Thyroid	Thyroid	71996	1506	849	1.18	56.37	1.79	7 (2–26)
Breast	All breast	317800	22087	5391	1.70	24.41	11.34	11 (3–27)
	HR+/HER2−	214981	11133	2474	1.15	22.22	5.20	14 (4–31)
	HR+/HER2+	33561	3683	836	2.49	22.70	1.76	17 (5–31)
	HR−/HER2+	14343	2023	534	3.72	26.40	1.12	11 (3–25)
	HR−/HER2−	33931	2648	886	2.61	33.46	1.86	8 (2–15)
	Unknown	20984	2600	663	3.16	25.50	1.39	4 (1–19)
GI	Esophagus	18635	6886	1899	10.19	27.58	3.99	3 (1–8)
	Stomach	32352	8431	1707	5.28	20.25	3.59	7 (2–19)
	Small intestine	9925	1965	231	2.33	11.76	0.49	5 (1–11.5)
	Colon and rectum	174311	38217	9138	5.24	23.91	19.22	8 (2–19)
	Colon	132385	29604	6491	4.90	21.93	13.65	9 (2–23)
	Rectum	41926	8613	2647	6.31	30.73	5.57	10 (4–21)
	Anus	8389	608	190	2.26	31.25	0.40	8 (4–15)
	Liver	22420	4259	1881	8.39	44.17	3.96	2 (0–5)
	Biliary tract	8687	3148	740	8.52	23.51	1.56	3 (1–7.5)
	Pancreas	37878	21924	4238	11.19	19.33	8.92	2 (1–6)
	Head of pancreas	17871	6939	1213	6.79	17.48	2.55	3 (1–7)
	Body of pancreas	4956	3265	653	13.18	20.00	1.37	3 (1–7)
	Tail of pancreas	6693	5139	983	14.69	19.13	2.07	2 (1–5)
	Unspecified pancreas	8358	6581	1389	16.62	21.11	2.92	2 (1–5)
	Other GI	7474	4007	813	10.88	20.29	1.71	2 (0–6)
GYN	Cervix	17644	1661	783	4.44	47.14	1.65	5 (2–12)
	Endometrium	74852	3785	2056	2.75	54.32	4.33	6 (2–14)
	Ovary	26483	3302	1329	5.02	40.25	2.80	11 (2–25)
	Other GYN	11173	869	452	4.05	52.01	0.95	7 (2–21)
GU	Kidney	62992	11951	5531	8.78	46.28	11.64	6 (2–16)
	Bladder	72767	3061	1116	1.53	36.46	2.35	3 (1–8)
	Prostate	289240	14981	1247	0.43	8.32	2.62	12 (4–26)
	Testis	15736	1668	1126	7.16	67.51	2.37	19 (7–44)
	Other GU	7413	1198	468	6.31	39.07	0.98	4 (2–9)
Bone	Bone	4458	844	531	11.91	62.91	1.12	12 (5–26)
Skin	Melanoma	99708	2973	1067	1.07	35.89	2.24	4 (2–10)
Sarcoma	Sarcoma	19397	3292	1630	8.40	49.51	3.43	7 (2–19)
Others	Others	20903	5972	1713	8.19	28.68	3.60	3 (1–9)
All	All	1525441	171844	47537	3.1	27.7	100	6 (2–16)

LM: lung metastases; GI: gastrointestinal cancer; GYN: gynecologic cancer; GU: genitourinary cancer; IQR: interquartile range.

## Data Availability

The datasets generated and/or analyzed during the current study are available in the Surveillance, Epidemiology, and End Results Program repository (https://seer.cancer.gov/data/).

## Abbreviations

LM: Lung metastases
SEER: Surveillance, Epidemiology, and End Results
OS: Overall survival
HR: Hazard ratio
IQR: Interquartile range
CTCs: Circulating tumor cells
GI: Gastrointestinal cancer
GYN: Gynecologic cancer.
